# Distance learning during COVID-19 lockdown: Impact on adolescents with specific learning disorders and their parents

**DOI:** 10.3389/fpsyt.2022.995484

**Published:** 2022-10-19

**Authors:** Andrea Battisti, Giulia Lazzaro, Cristiana Varuzza, Stefano Vicari, Deny Menghini

**Affiliations:** ^1^Child and Adolescent Neuropsychiatry Unit, Department of Neuroscience, Bambino Gesù Children's Hospital, IRCCS, Rome, Italy; ^2^Department of Human Science, LUMSA University, Rome, Italy; ^3^Department of Life Science and Public Health, Catholic University of the Sacred Heart, Rome, Italy

**Keywords:** SARS-CoV-2, quarantine, stress, academic achievement, dyslexia, dyscalculia, education

## Abstract

**Background:**

The transition of teaching from in-person to Distance Learning (DL) due to the COVID-19 pandemic led to negative effects on students' psychological wellbeing and academic achievement. The worst consequences have been experienced by students with so-called *special educational needs*, as well as by their parents. However, very little emphasis has been placed on the effects of DL in students with Specific Learning Disorders (SLD). The present work aimed to evaluate the effects of DL during the COVID-19 lockdown in Italian students with SLD and in their parents.

**Methods:**

An online survey was administered to 92 students with SLD and their parents after the COVID-19 lockdown. The survey consisted of four sections: participants' demographic information; perceived stress related to general aspects (i.e., social and family determinants) as well as specific aspects related to DL; attitudes and feelings toward DL; and academic grades before and after DL.

**Results:**

Students with SLD perceived stress mainly from social isolation/distancing and DL (*p* always ≤ 0.0001), especially from online classes and oral exams (*p* always ≤ 0.0001). Students who did not benefit from appropriate accommodations (i.e., individualized teaching and learning methods) during DL perceived 3 times more DL-related stress than those who used them as in-person learning (OR = 3.00, CI 95%: 1.24–7.28, *p* = 0.015). Girls perceived more stress from online lessons (OR = 0.40, CI 95%: 0.16–0.96, *p* = 0.04) and use of devices (OR = 0.33, CI 95%: 0.14–0.80, *p* = 0.015) than boys. Negative feelings (less motivation, reduced ability to understand lessons, interact, and stay focused) and positive feelings (less anxiety and more self-confidence with its own rate of learning) toward DL emerged. Higher academic grades also was observed after DL (*p* ≤ 0.0001). Lastly, strong and positive correlations emerged between students' and parents' perceived stress during DL (*p* always < 0.001).

**Implications:**

The present study prompts special considerations for students with special educational needs not only when providing conventional instruction, but especially when it is necessary to suddenly modify teaching approaches.

## Introduction

Starting from early 2020, governments in most countries introduced drastic and restrictive measures to reduce the spread of COVID-19 infection. The restrictions disrupted social, relational, working, and economic lives of people around the world—inevitably marking an epoch. Since that time, the detrimental consequences in terms of psychosocial wellbeing and mental health, largely due to prolonged social isolation (1) and forced cohabitation (2), have been widely documented.

Most of all, children and adolescents have suffered the worst consequences of the COVID-19 pandemic (1, 3, 4). The radical transformation of education was among the most challenging issues for young people and, consequently, their parents. As reported by the United Nations Educational Scientific and Cultural Organization, starting from March 2020, 107 countries have imposed school closures due to COVID-19 pandemic, affecting 862 million children and adolescents worldwide (5). School closures forced the disruption of in-person lessons, interfering with the daily routines of students and families with the activation of distance learning (DL). The move from in-person learning into online DL is unprecedented, and this transition has led to a readjustment of teaching methods using online platforms and videoconferencing tools to compensate for impossibility of in-person learning.

Generally, evidence documented that the experience of DL is usually associated with negative consequences in children and adolescents in terms of psychological wellbeing (5–8), learning outcomes and academic consequences (9–13). The findings also highlighted that the presence of associated medical conditions (14, 15), disabilities (16, 17), or neurodevelopmental disorders (18–20) in children and adolescents has markedly amplified the detrimental effects of DL—probably because of these already existing pre-conditions.

Surprisingly, little importance has been given to the effects of DL in children and adolescents who already have learning problems, such as specific learning disorders (SLD).

With a prevalence rate of 5%-15% worldwide, SLD are probably the most well-recognized neurodevelopmental conditions characterized by severe and enduring difficulties in the acquisition of reading (i.e., dyslexia) and/or math (i.e., dyscalculia) and/or writing (i.e., dysorthographia) in presence of adequate instruction and intellectual abilities (21). Such difficulties usually increase levels of stress and frustration at school and affect self-esteem with the onset of emotional and behavioral difficulties (22–25). During in-person learning, core difficulties of children and adolescents with SLD are mitigated to some extent by the implementation of individualized teaching and learning methods. In several countries, in order to provide the best conditions for learning and performing at school, students with SLD may benefit from specific regulatory prescriptions (e.g., Special Educational Needs and Disability Act, United Kingdom, 2001; Individual with Disabilities Education Act, USA, 2004; New regulations on Specific Learning Disorders in the school context, Italy, Law.170/2010). These prescriptions call for the application of appropriate accommodations, which include: the possibility of recording classroom explanations to facilitate home study, avoiding reading aloud and long dictations in class, using calculators and tables both at home and at school, scheduling oral exams specifying the content that will be required, the opportunity to consult visual maps during oral or written tests, and many others (New regulations on Specific Learning Disorders in the school context, Italy, Law.170/2010).

The DL imposed by the COVID-19 pandemic forced adjustments in teaching methods, especially for students with SLD, who already needed special supports and instruction tailored to their specific needs.

Only a few studies have focused on the impact of DL during the COVID-19 pandemic in students with SLD, particularly with dyslexia, examining the scenario in some European countries such as Italy (20, 26), Poland (27) and Spain (28, 29) considering personal and family emotional consequences (28, 29), perceptions of teaching quality (20, 27, 28), and consequences on academic achievement (26, 27).

Similar to findings on general population, when considering the emotional-behavioral consequences of DL, the little existing evidence on students with SLD highlighted a worsening of psychological wellbeing, with an increment of stress, anxiety, and depressive symptoms (28, 29).

The move from in-person learning into online DL has also posed additional challenges for families. Evidence generally documented that the experience of DL is usually associated with negative consequences for parents (28–31). In particular, studies have reported increased stress and difficulties in managing DL in parents of children and adolescents with pre-existing conditions such as Autism Spectrum Disorder [ASD; (19)], Attention Deficit Hyperactivity Disorder [ADHD; (18)], and Intellectual Disabilities [ID; (16)]. One possible reason for the increased parental stress during DL may lie in the absence of specialized care generally provided during school hours and the increased involvement of parents in the management of school activities. As well, parents of children and adolescents with SLD often feel overwhelmed during DL, with increased levels of stress, anxiety, and frustration also due to the reduced study autonomy of the children (28, 29, 32).

Considering perceptions of teaching quality, students with SLD seemed to have more difficulties in organizing and carrying out school activities during DL, experiencing a decrease in learning opportunities, greater difficulties in learning organization (e.g., adjusting the rate and time of work to their own need, ease of contact with the lectures), and a lack of support from teachers (20, 27–29).

With regard to academic achievement, students with dyslexia have been shown to have greater difficulty achieving their educational goals during DL regardless of school-grade (26, 27). Specifically, the study by Baschenis et al. (26) documented that around 61% of 65 Italian children and adolescents with dyslexia did not reach the level of reading speed generally achieved at the end of the school year [0.30 syllables/seconds for words; 0.15 syllables/seconds for non-words; (33)]. The availability of adequate accommodations or support services and the presence of a tutor during DL also did not positively influence the reading level achieved. Similarly, the study on Polish undergraduate students (27) observed that more students with dyslexia or self-reported reading difficulties failed at least one exam after the DL period compared to typical readers. Similarly, although it is difficult to compare with due to different parameters, studies on general population showed a learning loss of about 3 percentile points or 0.08 standard deviations in reading, maths and writing during the COVID-19 pandemic in about 60% of students (11, 13).

In the literature briefly summarized above, the heterogeneity of SLD manifestations (e.g., dyscalculia; dysorthography; dyscalculia and dyslexia; etc.) has been overlooked, while only dyslexia has been considered. Therefore, a comprehensive overview on the effects of DL, including a sample composed by different combinations of SLD, should be considered. In addition, the application of specific regulatory prescriptions in SLD and also the DL consequences on parents have been underestimated.

In light of this, the aim of the present study was to provide an integrated perspective of students with SLD and their parents regarding the experience of DL during the Italian COVID-19 lockdown, taking into consideration multiple aspects (e.g., stress, emotional state and self-efficacy, performance). Specifically, the present study aimed to address the following Research Questions (RQs):

RQ1: (a) What COVID-19-related factors (i.e., forced cohabitation, social distancing, DL) were most challenging for students with SLD? (b) Could sociodemographic variables (i.e., gender and age) and school compliance with prescriptions for SLD have influenced perceived stress associated with COVID-19-related factors in students with SLD?
RQ2: (a) What DL-related factors (i.e., homework, online lessons, written tests, oral exams, and use of devices in support to DL) were most challenging for students with SLD? (b) Could sociodemographic variables (i.e., gender and age) and school compliance with prescriptions for SLD have influenced perceived stress associated with DL-related factors in students with SLD?
RQ3: What were the attitudes and feelings of students with SLD toward DL?
RQ4: What were the academic grades before DL vs. after DL?
RQ5: (a) What DL-related factors (i.e., supporting students during online lessons, during homework, concomitant daily home activities and/or smart-working) were most challenging for parents? (b) Could students' age and parents' sociodemographic variables (age and educational levels) have influenced parental perceived stress associated with DL-related factors?
RQ6: Is there a relation between students' and parents' perceived stress during DL?

## Materials and methods

### Participants

Adolescents were retrospectively selected from a broad database of the Child and Adolescent Neuropsychiatry Unit of the Bambino Gesù Children's Hospital (Rome), upheld by the direction of the Head of the Unit (S.V.), consisting of several hundred patients who were evaluated at the hospital according to the good clinical practices per international guidelines for neurodevelopmental disorders by experienced developmental psychiatrists, neuropsychologists, and speech therapists.

All the adolescents included in the study received a diagnosis of SLD (dyslexia and/or dysorthography and/or dyscalculia) according to the Fifth Edition of the Diagnostic and Statistical Manual for Mental Disorders (DSM-5) criteria (21), developmental history and a comprehensive clinical and neuropsychological examination.

In particular, adolescents met the criteria for dyslexia and/or dysorthography and/or dyscalculia when the performance (i.e., accuracy and/or speed level) was at least 1.5 standard deviations below the mean for school-age in the norm-referenced reading measures (34, 35) and/or norm-referenced writing measures (34, 36), and/or norm-referenced arithmetic measures (37).

Adolescents were also assessed for potential neurodevelopmental or neuropsychiatric comorbidities. The presence of developmental coordination disorder and/or dysgraphia was assessed throughout an extensive neuropsychological evaluation with appropriate norm-referenced tests (36, 38). Moreover, others neurodevelopmental (e.g., ADHD, Tic Disorders) and eventually psychopathological comorbidities (e.g., anxiety and mood disorders) were clinically ascertained according to developmental history, extensive clinical examination, and Kiddie-Sads present and lifetime version—Diagnostic and Statistical Manual of Mental Disorders 5 (39).

Criteria for inclusion in the study were as follows: (1) having a diagnosis of SLD (dyslexia and/or dysorthography and/or dyscalculia); (2) attending secondary schools (age range between 11 and 19 years old); (3) non-verbal Intelligence Quotient (nvIQ) ≥ 70 (±5 points allowing for measurement error).

The exclusion criteria were as follows: (1) a diagnosis of ASD or ID; (2) having received the first diagnosis of SLD after the COVID-19 lockdown; (3) non-participation in DL despite school closures (e.g., lack of electronic devices, inadequate internet connection, or school difficulties with organizing DL).

### Procedures

The survey began after the conclusion of the Italian COVID-19 lockdown, at the end of May 2020, and was addressed to adolescents who had performed the DL and their parents. After selecting potential participants and their families based on inclusion/exclusion criteria, research assistants informed adolescents as well as their parents about the ongoing project. When both agreed to participation, the research assistants sent them *via* email a link through which they would have access to the self-report online survey. A total of 171 families were contacted, with a return rate of 96 students with SLD (56.14%) and 106 parents (61.99%).

The web-based survey included two questionnaires, one version for adolescents (students' version) and one for parents (mothers or fathers; parents' version). To ensure data privacy, an access code was provided for each participant. Each students' version was coded by “even” number while their respective parents' version with “odd” number. This method allowed the research assistants to combine the two versions of the survey (students' and parents') without tracking any sensitive data (e.g., first or last name, date of birth, etc.) of the respondents.

Adolescents as well as their parents provided consent and assent, respectively, before proceeding to the survey. The information collected was used in anonymous and aggregated form, in compliance with the EU General Data Protection Regulation n. 679/2016 (D.gls. n.196/2003 modified by D.gls. n. 101 del 10.08.2018). All procedures were consistent with the Declaration of Helsinki ethical principles for research involving human subjects.

### Online survey

The students' version was composed of 28 multiple and non-multiple-choice questions. It consisted of four sections that investigated:

- *Sociodemographic information* (age, gender, residential area, presence and number of siblings, order of parentage, and educational level).
- *General Perceived Stress*. Adolescents were asked to rate the level of stress experienced during Italian COVID-19 lockdown (from March to May 2020) in three different life contexts (family, school, and social). Likert-scale responses ranging from 0 (no stressful) to 10 (very stressful) were used.
- *Perceived Stress during DL*. Adolescents were asked to rate the level of stress experienced during Italian COVID-19 lockdown (from March to May 2020) in five school activities during DL: homework, online lessons, written tests, oral exams, and use of devices in support to DL (e.g., laptops). Likert-scale responses ranging from 0 (no stressful) to 10 (very stressful) were used.
- *Attitudes and feelings*. Adolescents were asked to indicate their attitudes (e.g., attention, motivation, relationship with teachers) and feelings (e.g., sadness, loneliness, anxiety) in relation to DL experience compared to in-person learning. “True or false” responses were used.

The parents' version was composed of 18 multiple and non-multiple-choice questions. It consisted of five sections investigating:

- *Sociodemographic information* (age, gender, marital status, educational level, and occupation of both parents).
- *Adolescents' need for help during DL*. Parents were asked to indicate whether their children needed support for DL activities as well as who (such as mother, tutor, psychologist, speech therapist, etc.) provided support for DL. Multiple and non-multiple choice responses were used.
- *Application of regulatory prescriptions for SLD*. Parents were asked to indicate whether during DL, appropriate accommodations for SLD (New regulations on Specific Learning Disorders in the school context, Italy, Law.170/2010) have been applied as occurred during in-person learning. Multiple and non-multiple choice responses were used.
- *Perceived Stress during DL*. Parents were asked to indicate perceived stress in relation to supporting children during online classes and during homework as well as in relation to concomitant daily home activities and/or smart-working. Likert-scale questions ranging from 0 (no stressful) to 10 (very stressful) were used.
- *Adolescents' Academic Grades*. Parents were asked to indicate adolescents' grades before (from September to February 2020) and after (from March to May 2020) Italian COVID-19 lockdown in the following subjects: Italian, Math, and first foreign language (i.e., English).

For more details on the survey, see Supplementary materials.

### Statistical analyses

Since the assumptions of normality and homogeneity of variance were not met, non-parametric analyses were conducted.

In students with SLD, Friedman's ANOVAs were run within each of the following sections: General Perceived Stress (RQ1a), Perceived Stress during DL (RQ2a), and Academic Grades (RQ4). *Post-hoc* analyses were conducted using Wilcoxon signed-rank tests and Cohens' *d* was used as measure of effect size.

Regarding to RQ1b and RQ2b, logistic regression analyses were run to explore the association between the application of regulatory prescriptions for SLD (independent variable, 2 groups: “students who benefited from regulatory prescriptions for SLD” vs. “students who did not benefit from regulatory prescriptions for SLD”) and the stress levels of students with SLD (dependent variable, 2 categories: “low level” ranging from 0 to 5 and “medium-high level” ranging from 6 to 10). Other logistic regression analyses were used to examine the association between gender (independent variable) and the perceived stress.

For all logistic regressions, Odds Ratios (OR) and confidence intervals at 95% (CI 95%) were reported. To ensure the stability of the results, the limits of 95% of the bootstrapped distribution (R = 1,000) of beta coefficients bootstrapped percentile interval (95% BPI) were obtained.

In addition, regarding to RQ1b and RQ2b, non-parametric correlations (Spearman's Rank) were run to investigate the relation between students' age and stress measures (General Perceived Stress and Perceived Stress during DL). Where appropriate (General Perceived Stress, Perceived Stress during DL), the same analyses were conducted including students who did not have the parent survey associated (see Supplementary materials).

In parents, Friedman's ANOVA was run for the Perceived Stress during DL section (RQ5a). *Post-hoc* analyses were conducted by using Wilcoxon signed-rank tests and Cohens' *d* was used as measure of effect size.

Regarding to RQ5b, Spearman correlations were performed to explore the relation between students' age, parents' sociodemographic variables (age and educational levels) and parents' Perceived Stress during DL. Where appropriate (Academic Grades, parental Perceived Stress during DL), the same analyses were conducted including parents who did not have the student survey associated (see Supplementary materials).

Spearman correlations were also run between students' and parents' Perceived Stress during DL (RQ6).

The significance level was set at *p* < 0.05, and Bonferroni's correction for multiple comparisons was applied, when appropriate.

Analyses were run using SPSS for Windows (version 22.0; SPSS Inc., Chicago, IL).

## Results

### Sociodemographic information of students and parents

Of 96 students with SLD and 106 parents who completed the survey, a total of 92 student-parent dyads filled the survey.

Out of 92 adolescents (age in years: M = 14.4, SD = 1.94, range = 11–19; for more details, see Table 1), the majority (*n* = 87, 94.2%) were from central Italy (particularly Rome), specifically: Rome (*n* = 78, 84.8%), Frosinone (*n* = 1, 1.1%), Latina (*n* = 4, 4.3%), Rieti (*n* = 3, 3.3%), and Perugia (*n* = 1, 1.1%). The remaining students were from southern Italy (Catanzaro, *n* = 1, 1.1%; Lecce, *n* = 1, 1.1%; Potenza, *n* = 1, 1.1%) and northern Italy (Genoa, *n* = 1, 1.1%).

**Table 1:** Sociodemographic characteristics of students with SLD.

**Students**		**Number (%)**
Gender	Male	48 (52.2)
	Female	44 (47.8)
Siblings	Yes	74 (80.4)
	1	51 (68.9)
	≥2	23 (31.1)
	No	18 (19.6)
Order of parentage	First-born	22 (29.7)
	Second-born	38 (51.4)
	Third-born	12 (16.2)
	Fourth-born	2 (2.7)
Educational level	1st grade secondary school	31 (33.7)
	2nd grade secondary school	61 (66.3)
	Technical institution	20 (32.8)
	High school	41 (67.2)

Of the 92 adolescents, 12 had a diagnosis of dyslexia (13%), 7 presented dysorthography (7.6%), and only 4 were diagnosed with dyscalculia (4.4%). Forty-four adolescents (47.8%) had a combined diagnosis of dyslexia, dysorthography, and dyscalculia, while the remaining 25 (27.2%) presented different combination of SLD, specifically: 11 with dyslexia and dysorthography (12%), another 11 with dyslexia and dyscalculia (12%), and 3 with dysorthography and dyscalculia (3.3%). More than half of them (*n* = 57, 62%) benefited from the regulatory prescriptions for SLD during DL as occurred during in-person learning.

Most of students with SLD presented additional comorbid psychopathological (30 out of 92, 32.6%) or neurodevelopmental disorders (39 out of 92, 42.4%). Within psychopathological comorbidities, 22 (73.3%) presented anxiety disorders, 4 (13.3%) had mood disorders and the remaining had anxiety-depressive symptoms (*n* = 2, 6.6%) as well as emotional dysregulation (*n* = 1, 3.3%). Within neurodevelopmental comorbidities, most of adolescents presented developmental coordination disorder and/or dysgraphia (*n* = 24, 60.7%) and the remaining had a diagnosis of ADHD (*n* = 15, 16.3%; Combined presentation: *n* = 10, 9.2%; Predominantly inattentive presentation: *n* = 4, 4.4%; Predominantly hyperactive/impulsive presentation: *n* = 1, 1.1%), and transient Tic Disorder (*n* = 1, 3.3%).

Of the 92 parents (age in years: M = 49.1, SD = 4.75, range = 33–58), 84 mothers (91.3%) and 8 fathers (8.7%) answered the questionnaire (for more details, see Table 2). Of these, 47 parents (51.1%) reported that their children needed support during the DL period. In particular, 31 out of 47 parents (66%) supported their children during lessons and homework, while 13 adolescents (27.6%) were helped by a private teacher. Only 3 parents (6.4%) did not answer to the question.

**Table 2:** Sociodemographic characteristics of parents.

**Parents**		**Number (%)**
Marital status	Single	24 (26.1)
	Married	68 (73.9)
Educational level	1st grade secondary school	8 (8.7)
	2nd grade secondary school	37 (40.3)
	Bachelor's degree	21 (22.8)
	Master's degree	20 (21.7)
	PhD	6 (6.5)
Father's occupational status	Employed	83 (90.2)
	Unemployed	2 (2.2)
	Retired	1 (1.1)
	No answer	6 (6.5)
Mother's occupational status	Employed	73 (79.3)
	Unemployed	17 (18.5)
	No answer	2 (2.2)

### General perceived stress during COVID-19 lockdown in students with SLD (RQ1a and RQ1b)

When comparing the General Perceived Stress level among the family, school, and social contexts, Friedman's ANOVA revealed a significant difference [$\chi_{\left(2\right)}^{2}$ = 38.15, *p* ≤ 0.0001]. Wilcoxon signed-rank tests showed that students perceived more stress for the school context (5.82 ± 2.95) and social isolation/distancing (6.07 ± 3.28) than the family context (3.76 ± 3.13; respectively, school vs. family context: *Z* = 5.09, *p* ≤ 0.0001, Cohen's *d* = 1.25; social isolation/distancing vs. family context: *Z* = 4.94, *p* ≤ 0.0001, Cohen's *d* = 1.20). No significant difference emerged between school and social isolation/distancing (*Z* = 0.72, *p* = 0.47, Cohen's *d* = 0.15). After Bonferroni's correction for multiple comparisons (0.05/3), all statistical significances survived (*p* ≤ 0.017) (see also Supplementary Table S1).

Logistic regressions documented that gender was not associated with the amount of stress related to the family context [$\chi_{\left(1\right)}^{2}$ = 0.03, β_1_ = 0.08, 95% BPI: −0.82–1.04, OR = 1.08, CI 95%: 0.45–2.64, *p* = 0.86], nor to school context [$\chi_{\left(1\right)}^{2}$ = 2.88, β_1_ = −0.71, 95% BPI: −1.58–0.11, OR = 0.49, CI 95%: 0.21–1.13, *p* = 0.09], and nor to social isolation/distancing [$\chi_{\left(1\right)}^{2}$ = 2.74, β_1_ = −0.73, 95% BPI: −1.72–0.13, OR = 0.48, CI 95%: 0.20–1.16, *p* = 0.09].

No significant correlations emerged between age and General Perceived Stress, including family context, school context and social isolation/distancing (all *rho* between −0.02 and −0.11, *p* always > 0.32).

In addition, logistic regression showed that those who no longer benefited from regulatory prescriptions for SLD as in-person learning were 3 times more likely to be more stressed than those who benefited from prescriptions as in person-learning [$\chi_{\left(1\right)}^{2}$ = 6.20, β_1_ = 1.10, 95% BPI: 0.24–2.10, OR = 3.00, CI 95%: 1.24–7.28, *p* = 0.015].

### Perceived stress during distance learning in students with SLD (RQ2a and RQ2b)

Friedman's ANOVA showed a significant difference in stress perceived during DL for homework, online lessons, written tests, oral exams and use of devices [$\chi_{\left(4\right)}^{2}$ = 44.03, *p* ≤ 0.0001]. Students perceived more stress for online lessons compared to homework (*Z* = 3.73, *p* ≤ 0.001, Cohen's *d* = 0.84), written tests (*Z* = 4.26, *p* ≤ 0.001, Cohen's *d* = 0.99) and use of devices (*Z* = 5.55, *p* ≤ 0.001, Cohen's *d* = 1.42) but not than oral exams (*Z* = 1.65, *p* = 0.10, Cohen's *d* = 0.35), as shown by Wilcoxon signed-rank tests. After Bonferroni's correction for multiple comparisons (0.05/10), the aforementioned statistical significance survived (*p* ≤ 0.005). All the remaining comparisons are shown in Table 3 (see also Supplementary Table S1).

**Table 3:** Mean and standard deviation (SD) for distance learning perceived stress.

	**Questions^+^**	**M ±SD**	***Post-hoc* comparisons**
	**Stress related to…**		
	Homework (A)	5.26 ± 3.19	< B^∧^>E^∧^=C, D
	Online classes (B)	6.28 ± 3.08	>A^∧^, C^∧^, E^∧^=D
	Written tests (C)	4.87 ± 3.41	< B^∧^, D**>E*=A
	Oral exams (D)	5.65 ± 3.30	>C**, E^∧^=A, B
	Use of devices (E)	3.99 ± 3.33	< A^∧^, B^∧^, C*, D^∧^

^+^Likert-scale questions ranging from 0 (no stressful) to 10 (very stressful); ^*^p ≤ 0.05; ^**^p ≤ 0.01; ^∧^survived after Bonferroni's correction (*p* ≤ 0.005).

Logistic regressions documented that boys perceived less stress related to online lessons [$\chi_{\left(1\right)}^{2}$ = 4.40, β_1_ = −0.93, 95% BPI: −1.92 to −0.07, OR = 0.40, CI 95%: 0.16–0.96, *p* = 0.04] and to the use of devices [$\chi_{\left(1\right)}^{2}$ = 6.22, β_1_ = −1.10, 95% BPI: −2.07 to −0.25, OR = 0.33, CI 95%: 0.14–0.80, *p* = 0.015] compared to girls. No significant associations emerged between gender and stress related to homework [$\chi_{\left(1\right)}^{2}$ = 2.07, β_1_ = −0.61, 95% BPI: −1.48 to 0.23, OR = 0.55, CI 95%: 0.24–1.25, *p* = 0.15], nor to written tests [$\chi_{\left(1\right)}^{2}$ = 0.16, β_1_ = −0.17, 95% BPI: −1.01 to 0.67, OR = 0.85, CI 95%: 0.37–1.92, *p* = 0.69], nor to oral exams [$\chi_{\left(1\right)}^{2}$ = 0.21, β_1_ = −0.19, 95% BPI: −1.05 to 0.63, OR = 0.83, CI 95%: 0.36–1.88, *p* = 0.65].

No significant correlations emerged between age, and Perceived Stress during DL, including homework, online lessons, written tests, oral exams, and use of devices in support to DL (all *rho* between −0.03 and −0.13, *p* always > 0.21).

Moreover, no significant associations emerged between students who benefited from the regulatory prescriptions for SLD such as in-person learning and the perceived stress related to homework [$\chi_{\left(1\right)}^{2}$ = 0.34, β_1_ = −0.25, 95% BPI: −1.15 to 0.61, OR = 0.78, CI 95%: 0.33–1.81, *p* = 0.56], nor to online lessons [$\chi_{\left(1\right)}^{2}$ = 2.36, β_1_ = −0.69, 95% BPI: −1.62 to 0.19, OR = 0.51, CI 95%: 0.21–1.21, *p* = 0.13], nor to written tests [$\chi_{\left(1\right)}^{2}$ = 0.29, β_1_ = 0.23, 95% BPI: −0.62 to 1.11, OR = 1.26, CI 95%: 0.54–2.93, *p* = 0.59], nor to oral exams [$\chi_{\left(1\right)}^{2}$ = 0.18, β_1_ = 0.18, 95% BPI: −0.67 to 1.08, OR = 1.20, CI 95%: 0.51–2.80, *p* = 0.67], and nor to the use of devices [$\chi_{\left(1\right)}^{2}$ = 0.17, β_1_ = −0.19, 95% BPI: −1.18 to 0.72, OR = 0.83, CI 95%: 0.35–1.99, *p* = 0.68].

### Student attitudes and feelings toward distance learning in students with SLD (RQ3)

Table 4 shows the percentages of students with SLD who preferred learning during DL over in-person learning (items 2–7) and how they felt during DL vs. in-person learning (items 8–13).

**Table 4:** The percentages of students with SLD in favor of DL compared to in-person learning in terms of attitudes (items 2–7) and feelings (items 8–13).

**Items**			**Number (%)**
			**in favor of DL**
	1.	The amount of homework was less than when I went to school regularly.	47 (51.1)
Attitudes toward DL	2.	DL was better suited to my learning rate.	54 (58.7)
	3.	It was easier for me to understand the lessons than when I went to school regularly.	21 (22.8)
	4.	It was easier for me to intervene in front of other classmates than when I went to school regularly.	26 (28.3)
	5.	It was easier for me to ask teachers for clarifications than when I went to school regularly.	28 (30.4)
	6.	It was easier for me to get attention from teachers during lessons than when I went to school regularly.	23 (25)
	7.	Even when there were no lessons, it was easier for me to get attention from teachers than when I went to school regularly.	28 (30.4)
Feelings toward DL	8.	I think I did less well in the DL than when I went to school regularly.	55 (59.8)
	9.	It was easier for me to pay attention and focus on the lesson than when I went to school regularly.	12 (13)
	10.	Before DL, I was less anxious about homework and questions in school.	57 (62)
	11.	I felt sadder than when I went to school regularly.	44 (47.8)
	12.	I felt lonelier than when I went to school regularly.	40 (43.5)
	13.	I felt more motivated to study than when I went to school regularly.	24 (26.1)

Of 92, 54 (58.7%) students stated that DL was more suited to their learning rate compared to in-person learning (item 2). In contrast, most students pointed out that during in-person learning, it was easier to understand lessons (*n* = 71, 77.2%; item 3), to intervene in front of other classmates (*n* = 66, 71.7%; item 4), to ask teachers for clarifications (*n* = 64, 69.6%; item 5) and to get attention from teachers during (*n* = 69, 75%; item 6) or after lessons (*n* = 64, 69.6%; item 7) compared to DL.

In addition, most of students reported perceiving more self-efficacy (*n* = 55, 59.8%; item 8) and being less anxious (*n* = 57, 62%; item 10) during learning *via* DL compared to in-person learning.

In contrast, the majority of adolescents pointed out that during in-person learning, it was easier to pay attention and focus (*n* = 73, 79.3%; item 9) as well as they felt more motivated (*n* = 68, 73.9%; item 13) compared to DL.

Overall, out of 92, only 1 student answered the questions on DL consistently and positively and 6 students experienced DL in a totally negative way. The remaining 85 students answered both positively and negatively to the different questions about attitudes and feelings toward DL.

### Academic grades (RQ4)

Friedman's ANOVA revealed a significant difference before and after DL in academic grades in the subjects Italian, Math and English [$\chi_{\left(5\right)}^{2}$ = 30.29, *p* ≤ 0.0001]. Students with SLD obtained higher Academic Grades in all subjects after DL (Wilcoxon signed-rank tests) compared to before DL (respectively, before DL vs. after DL: Italian, 6.55 ± 0.87 vs. 6.87 ± 0.98, *Z* = 4.19, *p* ≤ 0.0001, Cohen's *d* = 0.97; Math, 6.48 ± 1.21 vs. 6.82 ± 1.22, *Z* = 3.47, *p* ≤ 0.001, Cohen's *d* = 0.78; English, 6.49 ± 1.16 vs. 6.79 ± 1.24, *Z* = 3.92, *p* ≤ 0.0001, Cohen's *d* = 0.90). After Bonferroni's correction for multiple comparisons (0.05/3), all statistical significances survived (*p* ≤ 0.017) (see Supplementary Table S1).

### Parental perceived stress during distance learning (RQ5a and RQ5b)

Friedman's ANOVA did not reveal a significant difference in the perceived stress related to supporting children during online lessons (4.32 ± 3.48), nor during homework (4.84 ± 3.38), nor to concomitant daily home activities and/or smart-working [4.69 ± 3.60; $\chi_{\left(2\right)}^{2}$ = 4.46, *p* = 0.11] (see also Supplementary Table S1).

Correlations analyses documented that age of adolescents was significantly and negatively related with parental stress in supporting children during online classes (*rho* = −0.26, *p* = 0.011), meaning that as adolescents' age decreased, parents perceived more stress. The same relation emerged between parental age and online classes support (*rho* = −0.24, *p* = 0.022), meaning that the younger the parents, the more stress they experienced. However, after applying Bonferroni's correction (*p* = 0.05/9 = 0.006), none of these significances survived.

No other significant correlations between parental perceived stress and parental educational level, adolescents' and parental age emerged (all *rho* between −0.02 and −0.19, *p* always > 0.07).

### Relation between students' and parents' perceived stress during distance learning (RQ6)

When analyzing parental stress and stress of adolescents during DL, we found that:

- The greater the general stress perceived by adolescents during DL and the greater the parental stress related to daily home activities and/or smart-working (*rho* = 0.42, *p* = 0.00003);
- The greater the stress of adolescents during homework and the greater the parental stress in supporting them during this activity (*rho* = 0.37, *p* = 0.0003);
- The greater the stress of adolescents during online lessons and the greater the parental stress in supporting them during this activity (*rho* = 0.37, *p* = 0.0003).

After Bonferroni's correction for multiple comparisons, all statistical significances of correlations survived (*p* = 0.05/9 = 0.006).

## Discussion

In the first half of 2020, one of the most drastic dispositions to contain the spread of COVID-19 contagions was the closure of all schools and universities worldwide.

The negative consequences of school closures in the daily lives of students (5–13) and families (4, 9, 31, 40) were particularly significant for those with so-called special educational needs (14–20).

The present study aimed at investigating the integrated perspective of students with SLD and their parents regarding the experience of DL during the Italian COVID-19 lockdown in terms of stress, attitudes and feelings toward DL, and academic grades.

Our results showed that students with SLD during COVID-19 lockdown perceived a higher level of stress from school and social isolation than stress in the family context. The current findings provide an opportunity to reflect on the consequences of DL for students with SLD on psychological wellbeing. Previous reports have emphasized that social distancing and the inability to freely engage with peers has represented arguably the most considerable stressor for adolescents (41, 42). The similar level of stress we found for school and social distancing can be interpreted as the effect, in both contexts, precisely of the inability to have relationships with peers both at school and on extracurricular occasions. Alternatively, we can hypothesize that for students with SLD adapting their educational needs in a new and unexplored way was as severe a burden as the impossibility of meeting peers for months.

Moreover, we found that students who no longer benefited from the regulatory prescriptions for SLD as in-person learning were 3 times more likely to be stressed than those who benefited from the prescriptions as in-person learning. Our results highlight the relevance of appropriate accommodations for students with SLD, not only to put them in the best conditions to learn and perform, but also to preserve their confidence in coping with school demands. Although different approaches to support educational support strategies for students with SLD exist worldwide, our findings are in line with other studies documenting a lack of consideration of special educational needs during DL (28, 29). We can postulate that teachers' difficulties in using new technologies and digital tools during pandemic (43), together with the effort to quickly adjust lessons in this new modality, might lead to less attention being paid to the educational needs of students with SLD.

Regarding the aspects that influenced stress during DL, students were more stressed in relation to online lessons and oral exams than the remaining elements. Our results are consistent with previous findings (26), in which both students with dyslexia and their parents described greater difficulties in attending online lessons and a worsening in oral exposition compared to controls. Similarly, students with dyslexia were found to struggle with online lessons, believing that their educational needs were not sufficiently considered by teachers (28, 29). In contrast, the use of technology contributed less to DL-related stress, as evidenced by the lowest stress score obtained in relation to the use of devices. Given the average age of our student cohort, we can assume that they had already achieved sufficient autonomy in the use of devices and videoconferencing platforms, so this aspect did not produce significant stress.

Furthermore, our results showed that boys and girls perceived stress related to various components of DL differently. Specifically, girls perceived more stress from online lessons and use of devices than boys. Extensive literature documented a higher incidence of stress, anxiety, and depressive symptoms in girls than boys during lockdown (44–46). Nevertheless, given the absence of previous studies that specifically investigated gender differences in students with SLD when coping with DL, further studies are needed to draw conclusions on this issue.

When considering attitudes and feelings toward DL, our results revealed a dual facet of DL. On the one hand, students believed that DL was disadvantageous compared to the in-person learning for some reasons. Especially, most of students indicated that during DL, it was harder to understand lessons, intervene in front of other classmates, ask teachers for clarifications, and get attention from teachers compared to in-person learning. Similarly, students pointed out that they had difficulty paying attention and concentrating during the DL and felt less motivated compared to in-person learning.

It is well-documented that students with SLD often present attention difficulties (47–50), and it is therefore possible to suppose that the demands of DL have implied considerable attentional effort for these students. Furthermore, it should be noted that DL inevitably reduces opportunities to receive appropriate attention from teachers and to interact, and this may have reduced students motivation, given previous studies demonstrating that students with SLD particularly benefit from cooperative learning activities (51, 52), which became impossible during the lockdown.

Nevertheless, more than 50% of students stated that DL fit better with their learning rate than in-person learning. In addition, when considering the emotional state related to DL, most of students reported that they perceived better performance and were less anxious while learning *via* DL than in-person learning.

These results could depend on lower performance demands, less stringent assessment standards, and more parental support during DL. In this respect, students with SLD may have experienced the DL as less demanding and more compliant with their difficulties.

To verify whether DL also affected academic achievement of students with SLD, we compared grades obtained before and after DL in three school subjects, such as Italian, Math and English. Our results indicated an overall improvement after DL in all three subjects. Several hypotheses could be postulated to interpret this improvement:

- Teachers might have a greater tolerance in assessing student performance due to the challenging and unprecedented period, and given the greater difficulty during DL to provide students with SLD with individualized support than in-person teaching. This could be supported by our findings on the less application of SLD prescriptions compared to in-person learning and on the less interaction with teachers (such as asking teachers for less clarification and receiving less attention from teachers compared to in-person learning);
- As mentioned above, parents had the opportunity to provide direct support during DL to their children's school activities, which was not possible during in-person learning. In line with this hypothesis, in several studies involving students with and without special educational needs, parents were considered as proxy educators during the lockdown (4, 32, 40);
- DL may have somehow facilitated students with SLD, making them more confident in their abilities and more likely to succeed than in-presence learning. This latter hypothesis may be supported by the fact that most students indicated that DL was more suited to their learning rate than in-person learning. In addition, the use of technological measures may have benefited them because it prevented them from performing certain activities in the traditional way, such as writing by hand or reading from the book, that are particularly demanding for students with SLD.

To the best of our knowledge, no study that has explored the effects of DL in students with SLD has evaluated academic grades before and after DL. In particular, only reading skills before and after DL were compared, observing less than expected progress (26). Moreover, a study on university students with SLD (27) evaluated the amount of exams passed during DL in comparison with those passed by typically readers, observing a worse performance only on the former. It is therefore difficult to compare our results, which depend on teachers' judgments, with those derived from comparing more objective measures, such as the amount of exams passed or the reading level achieved before and after DL. It is therefore too premature to argue that the DL had a positive or detrimental effect on academic achievement of students with SLD.

The present study was designed to provide an integrated perspective of the DL experience, considering not only the self-perception of students with SLD, but also of their parents.

In particular, we examined the stress level of parents who had to work at home and reconcile their personal activities with their children's demands for support during DL and homework.

Parents reported that their stress levels related to supporting their children during online classes, during homework, and during concomitant daily household and/or smart-working activities were similar. Instead, we had hypothesized that the balance between work demands and child support during DL and homework was an important stressor. It could be hypothesized that parents experienced co-presence with their children at home in a positive way, as it gave them the opportunity to spend time together and to support them more than is usually the case due to work commitments. Our results show, however, that parental stress went hand in hand with children's stress, and the greater the children's perceived stress during DL, homework and online classes, the greater the parents' stress. These results support previous findings, which showed that when parents assumed the role of proxy educators during school closure and DL, the psychological wellbeing of the family was disturbed (4, 40).

Our study had some limitations.

The first limitation was the small number of participants included in the study and their homogenous geographic area (Rome)—which did not allow us to draw definitive conclusions. Despite the large number of families reached, the need to combine the two versions of the survey (for parents and students) forced us to exclude many incomplete questionnaires.

Another limitation might be that we did not compare the impact of DL between students with SLD and students who present another neurodevelopmental disorder. This could have clarified whether the observed negative and positive aspects of DL are typical of students with SLD or common to students with other special educational needs.

Moreover, elements such as personality predisposition to be more anxious, more motivated, or more self-confident may have partly influenced the answers provided in the survey. It would have been even more informative to assess the relationship between students' characteristics and perceptions of the DL.

Finally, the present study did not generally assess parents' level of psychological wellbeing during the lockdown period. In fact, our questions focused specifically on the impact the DL had on parents and students. It would have been useful to have information on the parent-child relationship before school closures, to see how the DL experience and previous family interactions would have interplayed with each other.

Moreover, taking into account the general level of stress and emotional state of parents during the lockdown period could have been helped us to better understand the relation between parents' and students' experience about DL.

## Conclusion

The COVID-19 pandemic has affected everyone's life to some extent. Certainly, the heaviest cost has been paid by adolescents, who have seen their primary need, the school, challenged. The prolonged school closures and the spread of DL have brought out critical issues and strengths of alternative and technology-based teaching methods. In this context, our study offers further insights into the importance of considering the different needs of all students, including those with SLD. Given the uncertainty that still seems to characterize this pandemic phase, adopting appropriate strategies and planning teaching activities taking account of all students' needs could be a crucial challenge for the future.

## Data availability statement

The raw data supporting the conclusions of this article will be made available by the authors, without undue reservation.

## Ethics statement

Ethical review and approval was not required for the study on human participants in accordance with the local legislation and institutional requirements. Written informed consent to participate in this study was provided by the participants' legal guardian/next of kin.

## Author contributions

GL, CV, SV, and DM designed the study. AB, GL, and CV collected the data. AB, GL, and DM worked on data analyses. AB and GL drafted the manuscript, with support of DM and SV. DM and SV supervised the study. All authors contributed to the article and approved the submitted version.

## Funding

This work was supported by the Italian Ministry of Health with Current Research funds.

## Conflict of interest

The authors declare that the research was conducted in the absence of any commercial or financial relationships that could be construed as a potential conflict of interest.

## Publisher's note

All claims expressed in this article are solely those of the authors and do not necessarily represent those of their affiliated organizations, or those of the publisher, the editors and the reviewers. Any product that may be evaluated in this article, or claim that may be made by its manufacturer, is not guaranteed or endorsed by the publisher.
