# Supplementary material for: Distance learning during COVID-19 lockdown: Impact on adolescents with specific learning disorders and their parents
Source: Front Psychiatry. 2022 Oct 19;13:995484. doi: 10.3389/fpsyt.2022.995484 (PMC9627200; doi:10.3389/fpsyt.2022.995484)
Supplement: Supplementary file 1 [file Data_Sheet_1.docx]

**Supplementary Materials**

**The experience of Distance Learning during the Italian COVID-19 lockdown**

Dear Student,

in this moment of public health emergency due to the spread of the Covid-19 virus, we have been called to change our daily rhythms of life and modify our habits. The government's containment measures have forced us to spend most of our time at home, with our families, and to go out only in case of necessity.

The Bambino Gesù Children's Hospital in Rome is carrying out a research about the impact of Distance Learning (DL) experience in students and their parents during the quarantine imposed.

For this purpose, we are asking you to take a few minutes to complete the following questionnaire.

Your answers will be processed anonymously and are extremely important for the purposes of the research.

(*Follows information and consent to the processing of sensitive personal data*)

**1) Sociodemographic information**

1. How old are you?
2. Indicate your gender
3. Where do you live?
4. Do you have any siblings?
5. If yes, how many?
6. Are you a first-born, second-born, third-born, fourth-born, or more?
7. Indicate your educational level

**2) Rate from 0 (no stress) to 10 (high stress) concerning the stress you perceived in the first lockdown period (March 2020 to May 2020) in the following contexts:**

1. Family _____
2. Social _____
3. School _____

**3) For each of the following distance learning aspects, rate from 0 (no stress) to 10 (high stress) concerning the stress you perceived during the distance learning period (March 2020 to May 2020):**

1. Homework **_____**
2. Online lessons **_____**
3. Written tests **_____**
4. Oral exams **_____**
5. Use of devices **_____**

**4) Indicate whether the following sentences concerning the distance learning period (March 2020 to May 2020) are true or false for you (0 = false; 1 = true):**

1. The amount of homework was less than when I went to school regularly **_____**
2. DL was better suited to my learning rate **_____**
3. It was easier for me to understand the lessons than when I went to school regularly **_____**
4. It was easier for me to intervene in front of other classmates than when I went to school regularly **_____**
5. It was easier for me to ask teachers for clarifications than when I went to school regularly **_____**
6. It was easier for me to get attention from teachers during lessons than when I went to school regularly _____
7. Even when there were no lessons, it was easier for me to get attention from teachers than when I went to school regularly **_____**
8. I think I did less well in the DL than when I went to school regularly **_____**
9. It was easier for me to pay attention and focus on the lesson than when I went to school regularly **_____**
10. Before DL, I was less anxious about homework and questions in school **_____**
11. I felt sadder than when I went to school regularly _____
12. I felt lonelier than when I went to school regularly _____
13. I felt more motivated to study than when I went to school regularly _____

**The experience of Distance Learning during the Italian COVID-19 lockdown**

Dear Parent,

in this moment of public health emergency due to the spread of the Covid-19 virus, we have been called to change our daily rhythms of life and modify our habits. The government's containment measures have forced us to spend most of our time at home, with our families, and to go out only in case of necessity.

The Bambino Gesù Children's Hospital in Rome is carrying out a research about the impact of Distance Learning (DL) experience in students and their parents during the quarantine imposed.

For this purpose, we are asking you to take a few minutes to complete the following questionnaire.

Your answers will be processed anonymously and are extremely important for the purposes of the research.

(*Follows information and consent to the processing of sensitive personal data*)

**1) Sociodemographic information**

1. How old are you?
2. Indicate your gender
3. Indicate your educational level
4. Are you employed or unemployed?
5. If you are employed, please specify your profession
6. Are you married?

**2) Child’s need for help during DL**

1. Did your child need to be supervised during DL? **_____**
2. If yes, by whom?

**3) Have the specific regulatory prescriptions for SLD been applied for your child as during in-person** **learning?**

**🗆** Yes

**🗆** No

**4) Rate from 0 (no stress) to 10 (high stress) concerning the stress you perceived in the first lockdown period (March 2020 to May 2020) in the following areas:**

1. Supporting your child during online classes **_____**
2. Supporting your child during homework **_____**
3. Daily home activities and/or smart-working **_____**

**5) Academic achievement:**

1. Please indicate your child’s marks before (from September to February 2020) the Italian COVID-19 lockdown in the following subjects:
2. Italian **_____**
3. Maths **_____**
4. First foreign language (i.e., English) **_____**
5. Please indicate your child’s marks after (from March to May 2020) the Italian COVID-19 lockdown in the following subjects:
6. Italian **_____**
7. Maths **_____**
8. First foreign language (i.e., English) **_____**

| **Table S1**. Summary of Friedman’s ANOVA results for Students with SLD (RQ1a, RQ2a, RQ4) and their parents (RQ5a). | | | | | |
| --- | --- | --- | --- | --- | --- |
|  | **Questions^+^** | **M ± SD** | **Friedman’s**  **ANOVA-value_(df)_** | ***p*-value** | **Post-hoc comparisons** |
| **Students** | **General Perceived Stress during COVID-19 (RQ1a)** |  | χ^2^_(2)_ = 38.15 | ≤ 0.0001 |  |
|  | School context (A) | 5.82 ± 2.95 |  |  | > C^***  = B |
|  | Social isolation/distancing (B) | 6.07 ± 3.28 |  |  | > C^***  = A |
|  | Family context (C) | 3.76 ± 3.13 |  |  | < A^***, B^*** |
|  | **Perceived Stress during DL (RQ2a)** |  | χ^2^_(4)_ = 44.03 | ≤ 0.0001 |  |
|  | Homework (D) | 5.26 ± 3.19 |  |  | < E^^1^  > H^^1^  = F, G |
|  | Online classes (E) | 6.28 ± 3.08 |  |  | > D^^1^, F^^1^, H^^1^  = G |
|  | Written tests (F) | 4.87 ± 3.41 |  |  | < E^^1^, G**  > H*  = D |
|  | Oral exams (G) | 5.65 ± 3.30 |  |  | > F**, H^^1^  = D, E |
|  | Use of devices (H) | 3.99 ± 3.33 |  |  | < D^^1^, E^^1^, F*, G^^1^ |
|  | **Academic Grades (RQ4)** |  | χ^2^_(5)_ = 30.29 | ≤ 0.0001 |  |
|  | Italian (before DL) (I)  Italian (after DL) (I^1^) | 6.55 ± 0.87  6.87 ± 0.98 |  |  | I^1^ > I^*** |
|  | Math (before DL) (J)  Math (after DL) (J^1^) | 6.48 ± 1.21  6.82 ± 1.22 |  |  | J^1^ > J^*** |
|  | English (before DL) (K)  English (after DL) (K^1^) | 6.49 ± 1.16  6.79 ± 1.24 |  |  | K^1^ > K^*** |
| **Parents** | **Perceived Stress during DL (RQ5a)** |  | χ^2^_(2)_ = 4.46 | 0.11 |  |
|  | Supporting children during online lessons (L) | 4.32 ± 3.48 |  |  | = M, N |
|  | Supporting children during homework (M) | 4.84 ± 3.38 |  |  | = L, N |
|  | Concomitant daily home activities and/or smart-working (N) | 4.69 ± 3.60 |  |  | = L, M |
| ^+^Likert-scale questions ranging from 0 (no stressful) to 10 (very stressful); *p ≤ 0.05; **p ≤ 0.01; ***p ≤ 0.001; ^survived after Bonferroni’s correction (p ≤ 0.017); ^^1^survived after Bonferroni’s correction (p ≤ 0.005). | | | | | |

## Supplementary Results

## General Perceived Stress during COVID-19 lockdown in Students with SLD (RQ1a and RQ1b)

When comparing the General Perceived Stress level among the family, school, and social contexts, Friedman’s ANOVA revealed a significant difference (χ^2^_(2)_ = 37.74, *p* ≤ 0.0001). Wilcoxon signed-rank tests showed that students perceived more stress for school context (5.85 ± 0.30) and for social isolation/distancing (5.98 ± 0.33) than for family context (3.78 ± 0.32; respectively, school v*s* family context: *Z* = 5.30, *p* ≤ 0.0001, Cohen’s *d* = 1.29; social isolation/distancing v*s* family context: *Z* = 4.93, *p* ≤ 0.0001, Cohen’s *d* = 1.16). No significant difference emerged between school and social isolation/distancing (*Z* = 0.45, *p* = 0.65, Cohen’s *d* = 0.09). After Bonferroni’s correction for multiple comparisons (0.05/3), all statistical significance survived (*p* ≤ 0.017).

Logistic regressions documented that gender was not associated with the amount of stress related to family context (χ^2^_(1)_ = 0.28, *β*_1_ = 0.24, 95% BPI: -0.72–1.22, OR = 1.27, CI 95%: 0.53–3.04, *p* = 0.59), nor to school context (χ^2^_(1)_ = 2.73, *β*_1_ = -0.68, 95% BPI: -1.61–0.11, OR = 0.51, CI 95%: 0.22–1.14, *p* = 0.10), and nor to social isolation/distancing (χ^2^_(1)_ = 1.23, *β*_1_ = -0.47, 95% BPI: -1.32–0.39, OR = 0.62, CI 95%: 0.27–1.44, *p* = 0.27).

No significant correlations emerged between age and General Perceived Stress, including family context, school context and social isolation/distancing (all *rho* between -0.07 and 0.01, *p* always > 0.52).

## Perceived Stress during Distance Learning in Students with SLD (RQ2a and RQ2b)

Friedman’s ANOVA showed a significant difference in stress perceived during DL for homework, online lessons, written tests, oral exams and use of devices (χ^2^_(4)_ = 48.65, *p* ≤ 0.0001). Students perceived more stress for online lessons compared to homework (*Z* = 3.37, *p* ≤ 0.001, Cohen’s *d* = 0.73), written tests (*Z* = 4.44, *p* ≤ 0.001, Cohen’s *d* = 1.02) and use of devices (*Z* = 5.72, *p* ≤ 0.001, Cohen’s *d* = 1.44) but not than oral exams (*Z* = 1.72, *p* = 0.08, Cohen’s *d* = 0.36), as shown by Wilcoxon signed-rank tests. After Bonferroni’s correction for multiple comparisons (0.05/10), the aforementioned statistical significance survived (*p* ≤ 0.005). See Table S2.

---Table S2---

Logistic regressions documented that boys perceived less stress related to the use of devices (χ^2^_(1)_ = 5.19, *β*_1_ = -0.98, 95% BPI: -1.93– -0.17, OR = 0.38, CI 95%: 0.16–0.88, *p* = 0.025) compared to girls. No significant associations emerged between gender and stress related to homework (χ^2^_(1)_ = 2.67, *β*_1_ = -0.67, 95% BPI: -1.49–0.17, OR = 0.51, CI 95%: 0.23–1.15, *p* = 0.10), nor to written tests (χ^2^_(1)_ = 0.04, *β*_1_ = -0.08, 95% BPI: -0.94–0.72, OR = 0.92, CI 95%: 0.41–2.05, *p* = 0.84), nor to oral exams (χ^2^_(1)_ = 0.04, *β*_1_ = -0.09, 95% BPI: -0.86–0.76, OR = 0.91, CI 95%: 0.41–2.04, *p* = 0.82), nor to online lessons (χ^2^_(1)_ = 3.10, *β*_1_ = -0.76, 95% BPI: -1.60–0.12, OR = 0.47, CI 95%: 0.20–1.10, *p* = 0.08).

No significant correlations emerged between age, and Perceived Stress during DL, including homework, online lessons, written tests, oral exams, and use of devices in support to DL (all *rho* between -0.03 and -0.13, *p* always > 0.21).

## Student Attitudes toward Distance Learning in Students with SLD (RQ3)

Of 96, 56 (58.3%) students stated that DL was better suited to their learning rate compared to in-person learning (item 2). In contrast, most of students pointed out that during in-person learning, it was easier to understand lessons (n = 74, 77.1%; item 3), intervene in front of other classmates (n = 69, 71.9%; item 4), ask teachers for clarifications (n = 68, 70.8%; item 5) and get attention from teachers during (n = 72, 75%; item 6) or after lessons (n = 67, 69.8%; item 7) compared to DL.

In addition, most of students reported perceiving more self-efficacy (n = 56, 58.3%; item 8) and being less anxious (n = 58, 60.4%; item 10) while learning through DL compared with in-person learning.

In contrast, the majority of adolescents pointed out that during in-person learning, it was easier to pay attention and focus (n = 76, 79.2%; item 9) as well as they felt more motivated (n = 71, 74%; item 13) compared to DL.

See Table S3.

Overall, out of 96, only 1 student answered the questions on DL consistently and positively and 7 students experienced DL in a totally negative way. The remaining 88 students answered both positively and negatively to the different questions about attitudes and feelings toward DL.

---Table S3---

## Academic Grades (RQ4)

Friedman’s ANOVA revealed a significant difference before and after DL in academic grades across Italian, Math and English subjects (χ^2^_(5)_ = 35.41, *p* ≤ 0.0001). Students with SLD obtained higher Academic Grades in all subjects after DL (Wilcoxon signed-rank tests) compared to before DL (respectively, before DL vs after DL: Italian, 6.53 ± 0.83 v*s* 6.87 ± 0.94, *Z* = 4.68, *p* ≤ 0.0001, Cohen’s *d* = 1.00; Math, 6.41 ± 1.25 v*s* 6.80 ± 1.14, *Z* = 3.95, *p* ≤ 0.0001, Cohen’s *d* = 0.81; English, 6.47 ± 1.10 v*s* 6.75 ± 1.17, *Z* = 4.08, *p* ≤ 0.0001, Cohen’s *d* = 0.84). After Bonferroni’s correction for multiple comparisons (0.05/3), all statistical significance survived (*p* ≤ 0.017).

## Parental Perceived Stress during Distance Learning (RQ5a and RQ5b)

Friedman’s ANOVA showed a significant difference in the perceived stress related to supporting children during online lessons (4.18 ± 3.50), during homework (4.89 ± 3.51), and to concomitant daily home activities and/or smart-working (4.74 ± 3.66; χ^2^_(2)_ = 6.49, *p* = 0.04). Wilcoxon signed-rank tests showed that parents perceived more stress related to supporting children during homework than to supporting children during online lessons (*Z* = 2.36, *p* = 0.02, Cohen’s *d* = 0.46), while no significant difference emerged between parents’ stress related to supporting children during homework and to concomitant daily home activities and/or smart-working (*Z* = 0.47, *p* = 0.64, Cohen’s *d* = 0.09), nor between parents’ stress related to supporting children during online lessons and to concomitant daily home activities and/or smart-working (*Z* = 1.41, *p* = 0.16, Cohen’s *d* = 0.27). After Bonferroni’s correction for multiple comparisons (0.05/3), the significance emerged did not survive (*p* ≤ 0.017).

Correlations analyses between parental age and parental perceived stress documented that parental age was significantly and negatively related to parental stress in supporting children during online classes (*rho* = -0.20, *p* = 0.04), meaning that meaning that the younger the parents, the more stress they experienced. However, after applying Bonferroni’s correction (0.05/3), the significance emerged did not survive (*p* ≤ 0.017).

No further significant correlations between parental perceived stress, parental age, and parental educational level (all *rho* between -0.01 and -0.17, *p* always > 0.08).

Table S2. Mean and standard deviation (SD) for Distance Learning Perceived Stress.

|  | **Questions^+^** | **M ± SD** | **Post-hoc comparisons** |
| --- | --- | --- | --- |
| **Students** | Stress related to… |  |  |
|  | Homework (A) | 5.41 ± 3.21 | < B^  > E^  = C, D |
|  | Online classes (B) | 6.31 ± 3.07 | > A^, C^, E^  = D |
|  | Written tests (C) | 4.90 ± 3.40 | < B^, D**  > E*  = A |
|  | Oral exams (D) | 5.69 ± 3.28 | > C**, E^  = A, B |
|  | Use of devices (E) | 4.02 ± 3.35 | < A^, B^, C*, D^ |

^+^Likert-scale questions ranging from 0 (no stressful) to 10 (very stressful); **p* ≤ 0.05; ***p* ≤ 0.01; ****p* ≤ 0.001; ^survived after Bonferroni’s correction (*p* ≤ 0.005).

| **Items** | | | **Number (%)**  **in favour of DL** |
| --- | --- | --- | --- |
|  | 1. | The amount of homework was less than when I went to school regularly. | 48 (50) |
| **Attitudes towards DL** | 2. | DL was better suited to my learning rate. | 56 (58.3) |
|  | 3. | It was easier for me to understand the lessons than when I went to school regularly. | 22 (22.9) |
|  | 4. | It was easier for me to intervene in front of other classmates than when I went to school regularly. | 27 (28.1) |
|  | 5. | It was easier for me to ask teachers for clarifications than when I went to school regularly. | 28 (29.2) |
|  | 6. | It was easier for me to get attention from teachers during lessons than when I went to school regularly. | 24 (25) |
|  | 7. | Even when there were no lessons, it was easier for me to get attention from teachers than when I went to school regularly. | 29 (30.2) |
| **Feelings toward DL** | 8. | I think I did less well in the DL than when I went to school regularly. | 40 (41.7) |
|  | 9. | It was easier for me to pay attention and focus on the lesson than when I went to school regularly. | 20 (20.8) |
|  | 10. | Before DL, I was less anxious about homework and questions in school. | 38 (39.6) |
|  | 11. | I felt sadder than when I went to school regularly. | 44 (45.8) |
|  | 12. | I felt lonelier than when I went to school regularly. | 42 (43.8) |
|  | 13. | I felt more motivated to study than when I went to school regularly. | 25 (26) |

Table S3. The percentages of children and adolescents with SLD in favour of LD compared to in-person learning in terms of attitudes (items 2-7) and feelings (items 8-13).
